# Novel perspectives for SARS-CoV-2 genome browsing

**DOI:** 10.1515/jib-2021-0001

**Published:** 2021-03-16

**Authors:** Visam Gültekin, Jens Allmer

**Affiliations:** Ekofan Soğutma, Mugla, Turkey; Hochschule Ruhr West, Institute for Measurement Engineering and Sensor Technology, Medical Informatics and Bioinformatics, Mülheim an der Ruhr, Germany

**Keywords:** COVID-19, entropy, genome browser, microRNA, secondary structure, TRS, variation

## Abstract

SARS-CoV-2 has spread worldwide and caused social, economic, and health turmoil. The first genome assembly of SARS-CoV-2 was produced in Wuhan, and it is widely used as a reference. Subsequently, more than a hundred additional SARS-CoV-2 genomes have been sequenced. While the genomes appear to be mostly identical, there are variations. Therefore, an alignment of all available genomes and the derived consensus sequence could be used as a reference, better serving the science community. Variations are significant, but representing them in a genome browser can become, especially if their sequences are largely identical. Here we summarize the variation in one track. Other information not currently found in genome browsers for SARS-CoV-2, such as predicted miRNAs and predicted TRS as well as secondary structure information, were also added as tracks to the consensus genome. We believe that a genome browser based on the consensus sequence is better suited when considering worldwide effects and can become a valuable resource in the combating of COVID-19. The genome browser is available at http://cov.iaba.online.

## 1 Introduction

Coronaviridae are a family of positive-strand RNA viruses that infect a wide range of hosts, including humans, livestock, and companion animals, causing human health problems, economic and animal welfare impacts [1]. COVID-19, caused by the coronavirus (CoV) SARS-CoV-2, has emerged as a real threat to society, putting a halt on everyday life and markets alike. SARS-CoV-2 likely emerged in China, presumably shortly before December 2019, and has subsequently spread globally, causing millions of infections and several hundred thousand deaths [2]. While varying among countries, the death toll is higher than was initially expected, and existing drugs do not specifically target SARS-CoV-2. Coronaviruses are among the viruses causing recurring infections such as the common cold where they were first described [1]. We currently witness a pandemic caused by a coronavirus of zoonotic origin, far surpassing the common cold’s effects. Due to the zoonotic origin, no prior immunity is inherent in humans, and no vaccines are available. Whether any antivirals are effective against SARS-CoV-2 is a matter of urgent research. Vaccines are being rolled out already, but their effectiveness and the immunity duration need to be determined. However, there seems to be no long-term immunity against coronaviruses causing the common cold with recurring infections throughout life. All CoV replicate in the cytoplasm of the host cell. Following attachment (ACE2) and uptake into the cell, the viral genome, which resembles cellular mRNA, is translated, leading to the production of the viral RNA synthesis machinery within the cytoplasm. This similarity to cellular RNA enables the replication of the virus and the production of sub-genomic viral mRNAs. Therefore, CoV infected cells contain numerous different viral RNA species.

Advances in technology have been serving all the science fields, and genomics is not an exception. Since the first announcement of sequencing technologies, scientists have been sequencing organisms ranging from the simplest forms of life to the most complex species [3]. The sequencing efforts also led to derivative studies; e.g., comparative genomics studies, coding sequence predictions, gene modeling, pathway analysis, and gene ontology studies. One challenge is combining the various information and presenting them to afford knowledge discovery. Genome browsers are designed for this exact task, enabling researchers to integrate all available data, extract and summarize information by visually browsing genomes. Genome browsers provide the opportunity to intuitively browse whole genomes, search and display specific regions down to a single base and see much of the available knowledge in context. Alongside the genome of interest, supporting annotations can be displayed separately, preserving the ability of browsing sequence data. These pre-computed and pre-generated annotations are defined as tracks and one can integrate many tracks such as gene models, RNA predictions, expression profiles. Following the announcement of the draft of the human genome assembly the UCSC genome browser was made available and still is one of the most used genome browsers along with the ensembl genome browser and NCBI’s genome data viewer as most notable and mainstream genome browsers [4]. These browsers offer a vast amount of data for many organisms and enable researchers to perform even cross-species analyses. Alongside these genome browsers, there are many others, some with a species-specific focus but including more annotations and perhaps even manually validated data [5].

A number of genome browser instances for the SARS-CoV-2 genome have become available, for example at UCSC [6], Ensembl [7], and NCBI (https://www.ncbi.nlm.nih.gov/nuccore/NC_045512.2). JBrowse, a novel web-based genome browser, which is used in this study to display genome information, provides a view of various data collected from other sources such as UCSC (http://covid19.jbrowse.org/).

All these instances of genome browsers for SARS-CoV-2 pick one genome and provide similar types of tracks. We aimed to provide additional information but chose not to use a resource with one specific underlying genome. In order to allow a more holistic view, we decided to create a consensus genome and use that as the base of our genome browser instance. Furthermore, presenting many tracks with slightly different genome assemblies appeared convoluted to us. Therefore, we chose to summarize the differences among the about 100 genomes in a single track. Obviously, we needed to translate the available gene annotations to this genome. Apart from these tracks we offer transcription regulating sequences (TRS) information which is a crucial component for CoV for the translation of sub-genomic parts of its genome. In addition to that, we included predicted miRNAs of SARS-CoV-2 and predicted targets of known human miRNAs and the bonding status of the RNA secondary structure. We make all information available to build the genome browser in the supplementary data and created an instance available on the web: http://cov.iaba.online We hope that the novel tracks we present here will also be included in other resources in the future, and that this genome browser for SARS-CoV-2 will provide researchers with novel vantage points at the available data.

## 2 Materials and methods

### 2.1 Consensus genome sequence

Sah et al. assembled the Coronavirus genome from a Nepalesian in early 2020 [8]. This sequence has been deposited in GenBank under the accession number MT072688 and at the GISAID EpiCoV newly emerging coronavirus SARS-CoV-2 platform under identifier EPI_ISL_410301. Since then hundreds of full length assemblies have become available. It was our aim to have an internationally representative genome sequence. Therefore, we chose to create a multiple sequence alignment of several genomes and use the consensus sequence from the alignment as the base for the genome browser. The NCBI virus variation database was used to select full length SARS-CoV-2 sequences. The following 102 genomes were selected: gb|MT198651, gb|MT198652, gb|MT198653, gb|MT020781, gb|MT163721, gb|MT039890, gb|MT163716, gb|MN988713, gb|MT184911, gb|MT093571, gb|MT184910, gb|MT019530, gb|MT123293, gb|MT123291, gb|MT192765, gb|MT184908, gb|MT184913, gb|MT039888, gb|MT012098, gb|MT126808, gb|MT066156, gb|MT159712, gb|MT027063, gb|MT027062, gb|MT007544, gb|MT019529, dbj|LC529905, gb|MT159705, gb|MT159722, gb|MN996531, gb|MT123290, dbj|LC528233, gb|MN996529, gb|MT066176, gb|MN994468, gb|MT159718, gb|MT027064, gb|MT159717, gb|MT159720, gb|MT121215, gb|MT192773, gb|MT192772, gb|MN996527, gb|MT072688, gb|MT188340, gb|MT093631, gb|MT159716, gb|MT039887, gb|MT019533, gb|MT106053, gb|MT159715, gb|MT019531, gb|MT184912, gb|MT118835, gb|MT159707, gb|MT159708, gb|MT159709, gb|MT044258, gb|MT192759, gb|MT039873, gb|MN988668, gb|MN988669, gb|MN996530, gb|MN996528, gb|MN908947, gb|MT019532, gb|MT159721, gb|MT184909, gb|MT159711, gb|MT159710, ref|NC_045512, gb|MT159719, gb|MT159713, gb|MT159714, gb|MT184907, gb|MT159706, gb|MN994467, gb|MT044257, gb|MT188341, gb|MT188339, gb|MT163717, gb|MT163718, gb|MT163720, gb|MT152824, gb|MT163719, gb|MT106054, gb|MT050493, gb|MT123292, gb|MT049951, gb|MT135043, gb|MT135044, gb|MT135042, gb|MT135041, gb|MN975262, gb|MT106052, gb|MN997409, gb|MN938384, emb|LR757995, gb|MT066175, gb|MN985325, gb|MT020881, gb|MT020880. The facilities at NCBI were also used to align the selected genomes. The alignment was used to create a consensus sequence with the most abundant nucleotide at each position. The resulting consensus sequence was named: SARS-CoV-2-consensus-v1-102genomes. This sequence differs from the Wuhan reference sequence which is widely used. We make the sequence available as a supplement with the name SARS-CoV-2-consensus-v1-102genomes.fasta.

### 2.2 NCBI gene annotation

The gene annotation for NC_045512.2 was retrieved from NCBI. Since the underlying genome is not identical with our consensus genome, the genes were mapped to the consensus using blastn. All genes could be mapped with minimal differences. The translated gene annotation is seen in the SARS-CoV-2 CDS track and can be downloaded.

### 2.3 Base variation

There are variations among the selected genomes which can be visualized. This can be achieved on a per genome basis. However, we believe an overview in one track to be very informative. For that a sequence profile was generated from the alignment and the results are presented in the Variation track. In order to provide a quick assessment of the variation at each locus, we calculated the Entropy which is defined as follows:

Entropy=∑−p(bi)*log2(p(bi))

where *b* is the observed frequency of one of the nucleotides and the gap. Thus no entropy, i.e., the same nucleotide in all sequences of the locus leads to an entropy of zero, whereas an equal distribution of all nucleotides and the gap leads to an entropy of 2.32. The observed values were scaled to be between 0 and 100 and visualized in one track entitled variation entropy. Additionally, we provide tracks for all 102 sequences used to create the consensus genome.

### 2.4 Transcription regulating sequence

The transcription regulating sequence (TRS) motif is an important feature for Coronavirus transcription [9] and, therefore, all matching TRSs are shown in the TRS track. Transcription is a bit different from eukaryotes and involves folding back of the RNA to initiate mRNA transcription from mRNA. Especially for subgenomic mRNA which is sub-selected involving TRS sequences. The motif sequence (CUAAACGAA) was taken from Xu et al. [10] and then matched against the consensus genome using blastn (word size: 4). All matches were added to a gff file and displayed regardless of their directionality. The gff file underlying the TRS track is available as a supplement (TRS.gff).

### 2.5 MicroRNAs

Putative microRNAs encoded in the Coronavirus genome were predicted by Sacar Demirci and Adan [11] using iZmiR [12], a state of the art miRNA prediction tool. These microRNAs were not predicted for the consensus genome and were therefore translated to the consensus genome by matching them with blastn to their closest location. All predictions are available for presentation in the predicted microRNAs track regardless of their confidence, not only the selected ones presented in the preprint by Sacar Demirci and Adan.

### 2.6 MicroRNA targeting

All currently known human miRNAs were submitted to psRNATarget [13] along with a coronavirus genome (NCBI accession: MT121215). The default options of psRNATarget were accepted without changes.

### 2.7 Secondary structure

RNA can form a secondary structure via base pairing. We used RNAFold [14] for the prediction of secondary structure from the primary RNA sequence. Secondary structure prediction is computational expensive when considering large sequences such as the complete viral genome. Interaction with other molecules or the genome with a copy of itself are also not modeled. Furthermore, local structures vary when predicted for regions of the genome or the complete genome. Therefore, we predicted the structure for portions of the genome comprising 80, 160, 250, and 500 nucleotides, respectively. The portioning of the genome in said fragments was performed with an overlap half the size of the respective fragment size. Additionally, we folded the full length genome. The file Folded_80-160-250-500-complete4.txt contains all folds in the following order: 0: the consensus RNA sequence, 1: 80nt, 2: 80nt with 40nt overlap, 3: 160nt, 4: 160nt with 80nt overlap, 5: 250nt with 6: 250nt with 125nt overlap, 7: 500nt, 8: 500nt with 250nt overlap, and 9: the fold of the complete RNA sequence. JBrowse does not have an available track type to display the dot bracket format representing RNA secondary structure. Therefore, we summarized the structural information on a per base level by counting how often the base is part of a bond. We display this information in the secondary structure bonding potential track. The data is part of the supplemental file mentioned above.

### 2.8 Genome feature translation

Any feature, such as the gene annotation from NCBI, that was not performed for the consensus genome directly needed to be translated. The underlying sequences for the features were retrieved and these sequences were compared to the consensus genome using blastn. An annotation was accepted when less than 1% nucleotides was different. Features made for the consensus genome such as the RNA secondary structure accessibility did not need any modification.

## 3 Results and discussion

Viruses are quick to adapt and thereby often evade treatment and, therefore, it may be useful to work with consensus sequences representing the virus instead of one arbitrarily chosen sequence. Even viruses, however, contain crucial information for their replication which is not subject to frequent change, such as regulatory sequences and structural motifs. These crucial parts of the viral genome could be targeted pharmaceutically.

### 3.1 Consensus sequence

**Figure 1:**
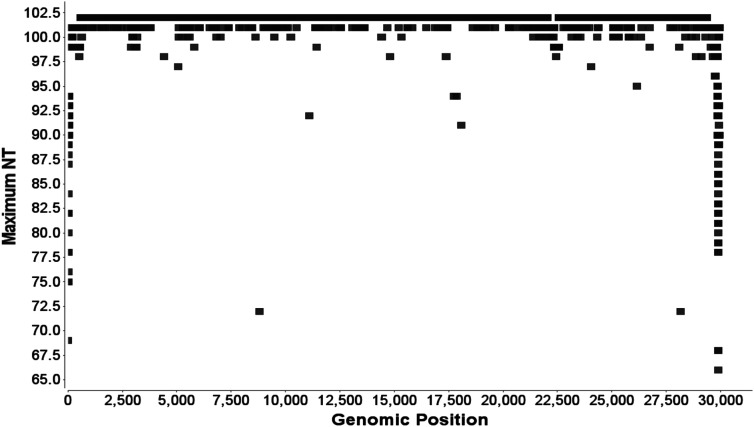
Base variation with genomic position. At each base the number for the maximum base is given. The absolute maximum is 102. Strongest variation would be 20 since 5 possible characters (A, C, G, T, and -) are considered at each position. The minimum of 66 is found at genomic position.

From the 102 selected full-length SARS-CoV-2 genome assemblies we created a multiple sequence alignment (Figure 1) using NCBI’s tools. The consensus sequence of this multiple sequence alignment is the nucleotide most frequent at each position. This consensus sequence forms the basis of the genome browser (supplemental file SARS-CoV-2-MSA.faln). The MSA has some variation on both extremes caused by frequent gaps (Figure 1). Within the sequence strong variation is rare and typically nucleotides are identical among assemblies (88%) or one assembly presents a difference (9%). The total length of the MSA is 29.959 bases. It contains 0.3% gaps, ∼32% U, ∼29% A, ∼18% C, and ∼19% G.

### 3.2 Variation track

**Figure 2:**
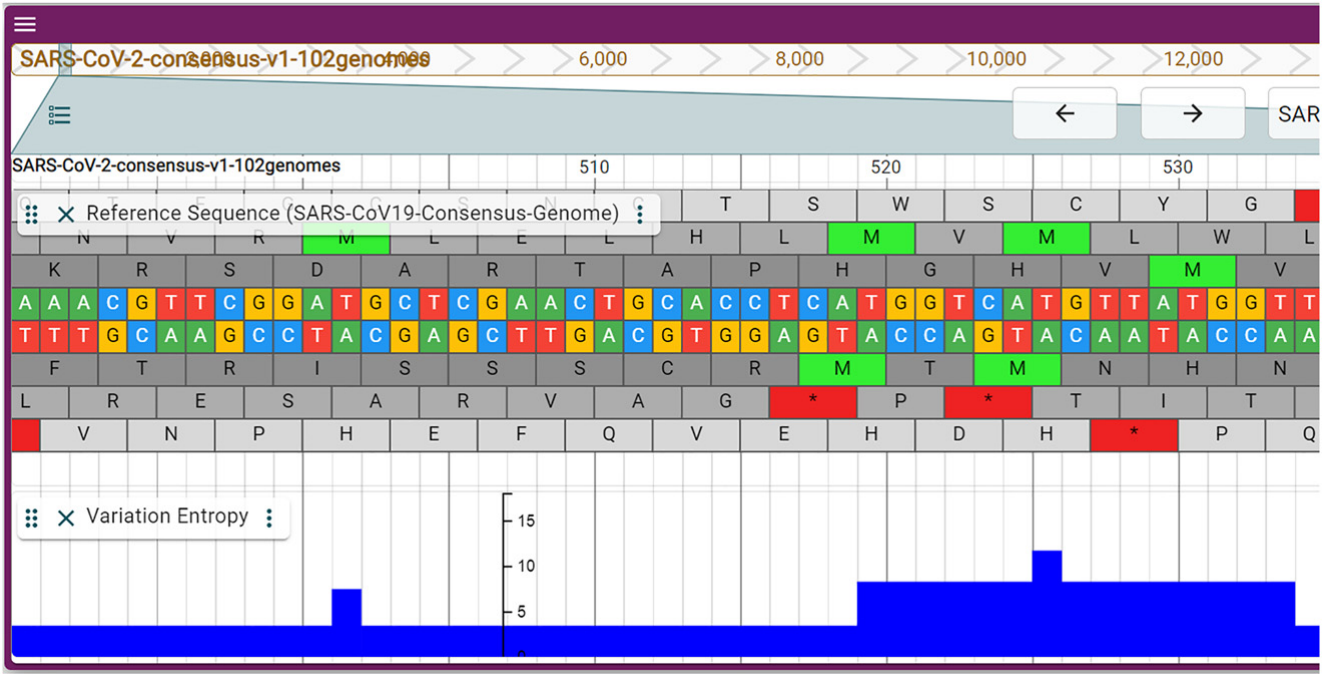
Zoom into the sequence and variation entropy track. The entropy at position 14.816 is 10.25 (blue bar). Zooming out reveals an inverted distribution compared to Figure 1.

As discussed above, there is some variation among genome assemblies. Typically, all genomes are aligned to the genome that forms the basis for the genome browser. The alignments are then shown in tracks and we provide the same functionality. Here we aimed to summarize the variation in a single track to offer a quick assessment of variation. In order to achieve this we calculated the entropy at each locus and display that in the variation entropy track. Low entropy means few differences among bases at the locus while a high entropy shows that the locus varies strongly among assemblies (Figure 2).

As can be expected from the assessment above, variation is highest at the extremes of the genome and very low in general. Some loci such as the one in Figure 2, may point to important differences among genomes. Here the variation is in orf1ab and has no overlaps with predicted miRNAs and doesn’t seem to be part of an important secondary structure or a TRS. The variation entropy track provides information at a quick glance. For interesting variations, the full alignments can be inspected in other tracks of the genome browser.

### 3.3 MicroRNAs and their targets

**Figure 3:**
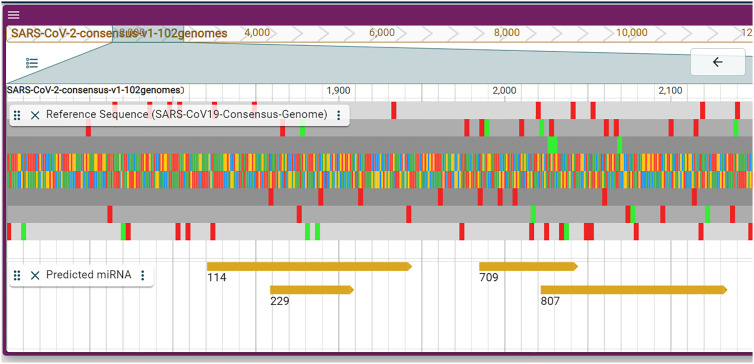
The track displaying the predicted miRNAs with four example miRNAs.

The predicted miRNAs by Sacar et al. [11] were mapped to the consensus genome using blastn. There were minimal differences in sequence for two predictions. All predictions are available for presentation in the Predicted MicroRNAs track regardless of their confidence and can be filtered using the genome browser interface (Figure 3).

Overall, 950 miRNAs have been predicted for SARS-CoV-2 by Sacar et al., 860 of which had a prediction score larger than 0.5. All predicted miRNAs were mapped to the consensus genome using blastn which led to ∼8500 matches. The matches were filtered by hairpin length (≥40) and similarity (less than 7 nucleotides difference were accepted as a match). A hairpin length of 40 was chosen since most human miRNAs in miRBase are longer than 40 nucleotides and because the virus has to rely on the human miRNA processing system. This left 312 matches in the consensus genome. Still, very unlikely candidates such as miRNAs 829 (score ∼0.30) and 106 (score ∼0.31) are included. Since these can be filtered in the genome browser, we chose to include such candidates. Sacar et al. also predicted human targets of confident miRNA predictions. These we cannot display in the SARS-CoV-2 genome browser. Conversely, targets of human miRNAs within the SARS-CoV-2 genome could be displayed. For that, we used the psRNATarget to predict targets for all known human miRNAs. 600 predictions were returned by psRNATarget. One important measure of psRNATarget is the expectation and the results fall in the range from two to five where five was the cutoff used during the prediction. The median expectation is 4.5 for the 600 predictions (Supplementary Figure 1). The lower the expectation, the better the prediction. Typically, we consider target predictions less or equal to two. Only two human miRNAs (hsa-miR-8066 and hsa-miR-193a-5p) have psRNATarget expect values worth considering, but they do not have the characteristic consecutive binding ín the 5′ region (bases 1|2-8). The prediction results are available in the supplementary file hsaMiRNAs_predicted_targets_in_CoV.txt. Thus, according to our data, humans cannot elicit a miRNA response against this coronavirus. We chose not to present a track with two questionable target predictions.

### 3.4 SARS-CoV-2 secondary structure

**Figure 4:**
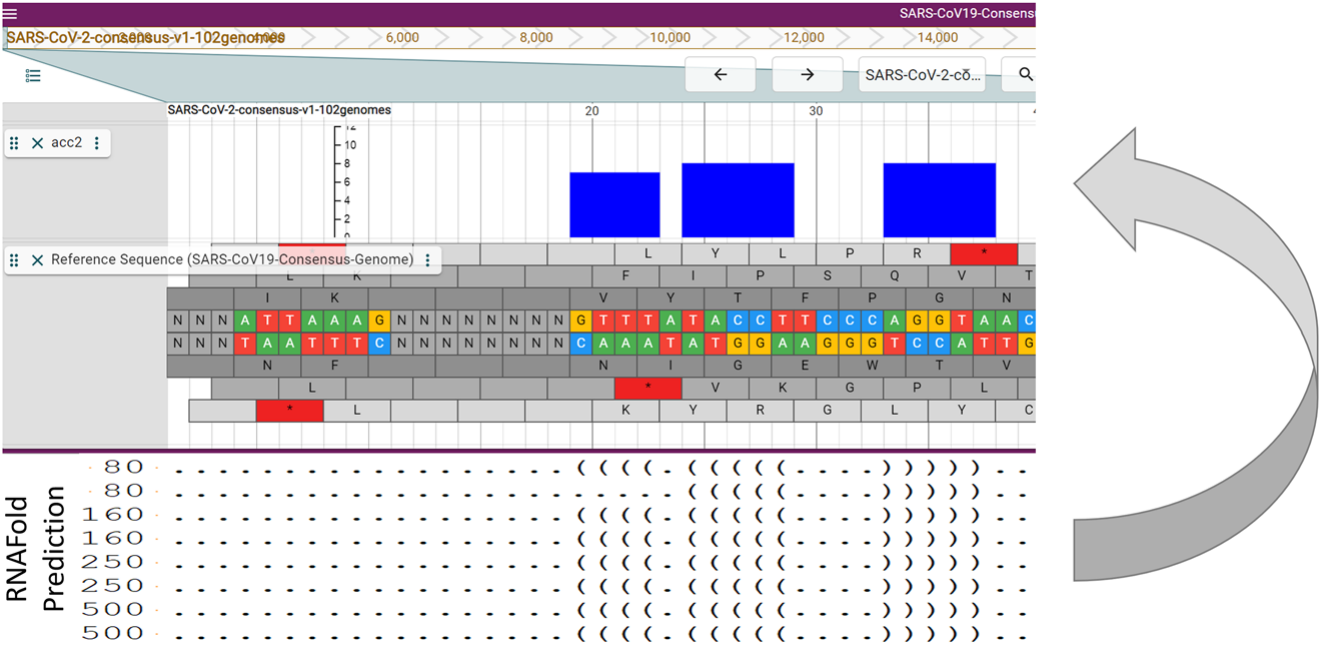
The first portion of the consensus genome (nucleotides 1 … 40). Below the genome browser are the predicted folds for the consensus genome from different sized fragments. The stacks in the secondary structure bonding potential track show the bonding status within the eight predicted folds. The RNAFold predictions consist of dots for no predicted bond and parentheses for bonds. Paired opening and closing parentheses form one bond. The bonding status ranges from zero (no predicted bond) to eight (bonds predicted for all fragments at that position).

RNA secondary structure is important for virus regulation, its interaction with the host, and may help reveal druggable targets [15], [16], [17], [18]. We predicted RNA secondary structure using RNAFold. Since for different fragment sizes of RNA different predictions are made, we chose 4 different fragment lengths and folded overlapping portions of the virus genome (Figure 4).

We used the eight structure predictions aligned to summarize the bonding potential for each base in the virus genome by counting the number of bonds at each position. Hence, bonding potential ranges from zero to eight in this study. Figure 4 has an example showing a part of a potential stem loop at the beginning of the SARS-CoV-2 genome. This track will help experimentalists to have a first understanding of the secondary structure and the bonding potential of the virus genome.

**Figure 5:**
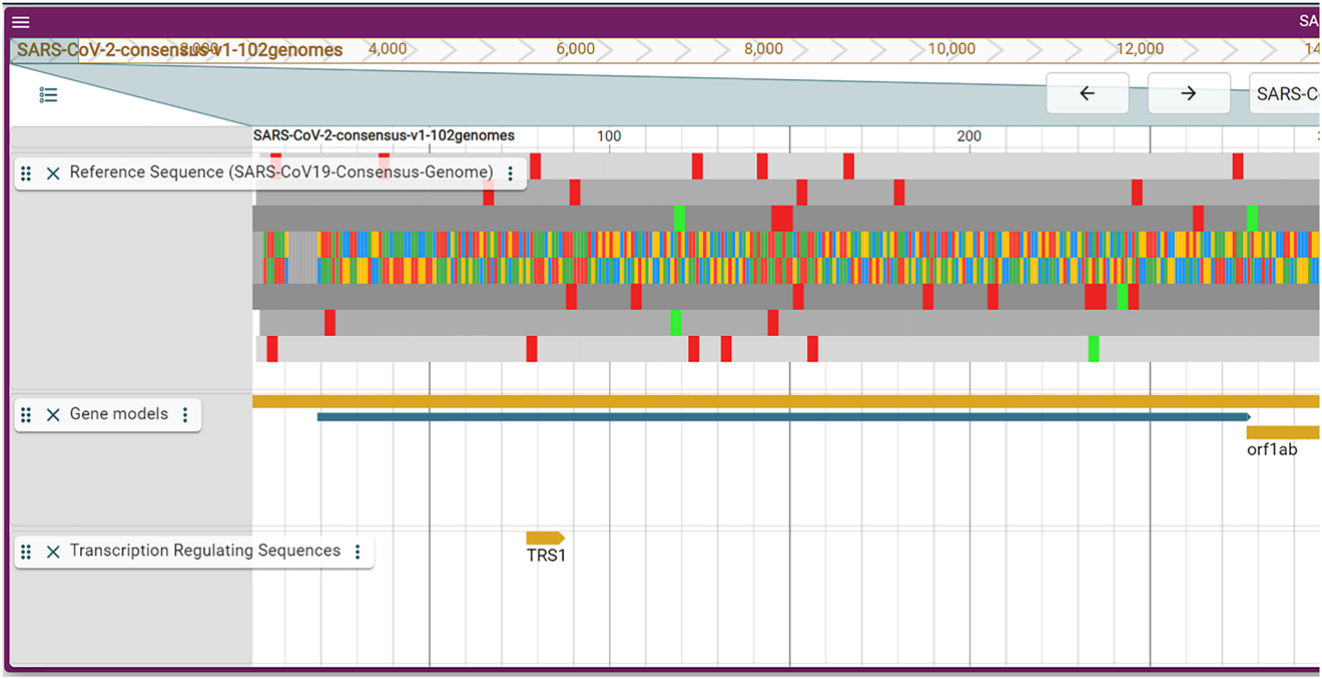
A portion of the SARS-CoV-2 genome with gene models and predicted TRS. The TRS track is on bottom and the gene models are located above. Zooming out, it can be seen that some TRS are in their expected locations (TRS 8-12). Other TRS may represent alternative products (TRS 4-7).

Along the same lines are transcription regulating sequences which are involved in producing sub-genomic RNAs for translation in Coronaviruses. TRS were predicted according to an available motif and mapped to the genome. TRS should be at the start of open reading frames and that is true for many of the predicted TRS (Figure 5).

However, some reside elsewhere and may not be functional although they could represent alternative gene products.

## 4 Conclusions

With the ongoing pandemic caused by SARS-CoV-2 it is important to gather as much information and view it from as many vantage points as possible. Here we present a genome browser based on a consensus genome instead of a single genome assembly in order to abstract from a single genome reference. This consensus sequence is accompanied by a track summarizing the variation and the multiple sequence alignment in another track. Additionally, we created a number of unique tracks which provide important information. These special tracks include the predicted SARS-CoV-2 miRNAs, the potential of each base to be part of an RNA secondary structure and predicted TRS. The genome browser is available at http://cov.iaba.online.

In the future, we aim to add expression information to enable an assessment of how parts of the genome are expressed on average. This information can prove useful in understanding CoV pathology and aid in developing drugs targeting CoV. We also aim to integrate community suggestions ranging from editing existing tracks to adding novel ones.
