# Supplementary figures and images for: Novel perspectives for SARS-CoV-2 genome browsing
Source: J Integr Bioinform. 2021 Mar 16;18(1):19–26. doi: 10.1515/jib-2021-0001 (PMC8035962; doi:10.1515/jib-2021-0001)

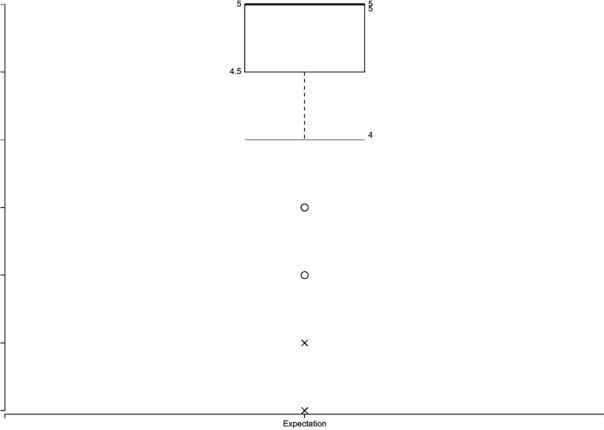

Supplement: Supplementary file 1 [file jib-18-20210001-s001.pdf]
